# Acute Watery Diarrhea Surveillance During the Rohingya Crisis 2017–2019 in Cox’s Bazar, Bangladesh

**DOI:** 10.1093/infdis/jiab453

**Published:** 2021-09-16

**Authors:** Md Taufiqul Islam, Ashraful Islam Khan, Zahid Hasan Khan, Nabid Anjum Tanvir, Faisal Ahmmed, Md Mokibul Hassan Afrad, Yasmin Ara Begum, Minjoon Kim, A S M Mainul Hasan, Maya Vandenent, M Salim Uzzaman, Tahmina Shirin, John D Clemens, Firdausi Qadri

**Affiliations:** 1 Infectious Diseases Division, International Centre for Diarrhoeal Disease Research Bangladesh (icddr,b), Dhaka, Bangladesh; 2 School of Medical Science, Griffith University, Gold Coast, Australia; 3 Health Section, United Nations Children’s Fund (UNICEF), Dhaka, Bangladesh; 4 Institute of Epidemiology Disease Control and Research (IEDCR), Dhaka, Bangladesh; 5 International Vaccine Institute, Seoul, Republic of Korea; 6 UCLA Fielding School of Public Health, Los Angeles, California, USA

**Keywords:** Bangladesh, cholera surveillance, Cox’s Bazar, Rohingya refugee

## Abstract

**Background:**

Forcibly Displaced Myanmar Nationals (FDMNs) fled into Cox’s Bazar, Bangladesh due to internal conflict. Considering the public health situation, a surveillance network was established to identify the enteric pathogens and early detection of cholera epidemics. The purpose of this manuscript is to report the clinical, epidemiological determinants of cholera and other enteric pathogens among hospitalized diarrheal patients from FDMNs and host community.

**Methods:**

A total of 11 sentinel surveillance sites were established around the camps in Ukhia and Teknaf Upazila, Cox’s Bazar. Rapid diagnostic testing was conducted for immediate detection of cholera cases. Stool samples were transferred to the Infectious Diseases Division, International Centre for Diarrhoeal Disease Research Bangladesh (icddr,b) laboratory for culture.

**Results:**

A total of 8134 participants with diarrhea were enrolled from 2017 to 2019: 4881 were FDMNs and 3253 were from the Bangladeshi host community. Among the FDMNs, the proportion of *Vibrio cholerae* was 0.7%, the proportion of enterotoxigenic *Escherichia coli* (ETEC) was 4.9%, and the proportion of *Shigella* was 1.5%. The distributions from host community were 1.2% *V cholerae*, 1.8% ETEC, and 1.1% *Shigella.* Similar risk factors have been identified for the diarrheal pathogens for both communities.

**Conclusions:**

This surveillance helped to monitor the situation of diarrheal diseases including cholera in refugee camps as well as in the neighboring host community. These findings lead policymakers to take immediate preventive measures.

Diarrhea is one of the leading causes of mortality across the globe. Cholera presents a substantial health burden in the developing world; it is endemic in Africa and Asia, and it has recently spread to America. An estimated 1.3 billion people worldwide are at risk of cholera; India and Bangladesh jointly constitute the largest share of this at-risk population [1]. Globally, an estimated 1.3 to 4.0 million cases and 21 000 to 143 000 deaths per year are attributed to cholera. The World Health Organization (WHO) acknowledges that worldwide, only 5%–10% of cholera cases are actually reported [2]. Experts suggest that approximately 66 million people are at risk, and each year 109 052 cholera cases along with 3272 deaths occur in Bangladesh [3].

The conflict in the Rakhine province of Myanmar in 2017 resulted a large influx of approximately 700 000 Forcibly Displaced Myanmar Nationals (FDMNs) in Cox’s Bazar, Bangladesh. Currently, the number stands at over 1 million [4]. Among the newly arrived displaced people, 60% are women and children. Public health conditions of the settlements of these individuals are poor, and they represent a risk for cholera outbreak [5]. In the recent past (eg, in Yemen, South Sudan, Haiti, and other countries), lack of Water, Sanitation and Hygiene (WaSH) and public health facilities have facilitated large epidemics with high numbers of cholera cases along with deaths [6]. Myanmar is also considered as endemic to cholera, and internally displaced person camps of Rohingyas in Northern Rakhine have regularly reported cases of severe acute watery diarrhea. As stated in the *Weekly Epidemiological Report*-2016 [7], the number of cholera cases reported to the WHO was 782 and 11 deaths, and the case-fatality rate was 1.4%. The International Co-ordination Group (ICG) of the WHO deployed 2 200 000 oral cholera vaccines (OCV) from the WHO emergency stockpile over the period of 2017 to 2019 in response to requests from the government of Bangladesh.

People living in these settlements are vulnerable to outbreaks of diarrheal disease including cholera as well as other infectious diseases. The Bangladeshi host population also lives in close proximity to these settlements. The FDMNs, especially women, children, and the elderly, have a variety of health problems. In Myanmar, women were deprived of reproductive healthcare and children suffered from malnutrition. Moreover, their immunization coverage was very low. Now, in Cox’s Bazar, they live in densely populated conditions in the camps with limited sources of clean water and sanitation, conditions that are favorable for an outbreak of cholera and other enteric diseases [8]. A nationwide surveillance of cholera has been carried out by the International Centre for Diarrhoeal Disease Research Bangladesh (icddr,b) in collaboration with the Institute of Epidemiology Disease Control And Research (IEDCR) in 22 sites (including Cox’s Bazar) in Bangladesh since 2014. The data showed that the proportion of cholera detection was 8.4% (among the tested samples) in Cox’s Bazar district even before the FDMNs arrived [9]. Soon after the influx of FDMNs, a diarrhea surveillance network was set up in and around the Rohingya camps at 2 subdistricts (Ukhiya and Teknaf) based on the procedures of the nationwide cholera surveillance. The surveillance was carried out for early detection of cholera epidemics. This study describes the clinical and epidemiological characteristics of enteric pathogens *Vibrio cholerae*, enterotoxigenic *Escherichia coli* (ETEC), and *Shigella* spp among FDMNs and the host population.

## METHODS

### Ethics Statement

We obtained informed consent (before the enrollment) from participants for collecting data and biological samples. For children younger than 18 years, informed consent was taken from participants and/or legal guardians. The surveillance protocol was approved by the Research Review Committee and Ethical Review Committee of icddr,b.

### Surveillance Sites

Surveillance was conducted in 9 sites in Ukhiya and 2 sites in Teknaf Upazila of Cox’s Bazar district, which is situated in Chattogram, Bangladesh. The areas include (1) Kutupalong and Balukhali makeshift camps in Ukhia and (2) Leda, Nayapara, and Shamlapur camps in Teknaf, which comprise a total of 34 camps. The Bangladeshi host population also lives in close proximity to the FDMNs. To provide a comprehensive picture of the epidemiology of pathogen-specific diarrhea among the FDMNs and host community, different health facilities (both government and private) around the camps were selected for the surveillance. Health facilities comprise primary healthcare centers, International Organization for Migration (IOM) hospitals, Médecins Sans Frontieres (MSF) clinics, other nongovernmental organization (NGO) hospitals and upazila health complexes.

### Operational Definition of Diarrhea

Any patient attending treatment facility with 3 or more loose or liquid stools within 24 hours or less than 3 loose/liquid stools causing dehydration was considered potentially eligible for the surveillance.

### Surveillance

At each site, trained staff were assigned to select the participants for enrollment. Patients with diarrhea who met the case definition and had no other severe comorbidity (eg, severe acute respiratory illness, acute cardiovascular symptoms, or severe acute neurological disorder) were selected for enrollment. Upon receiving consent, patients’ sociodemographic characteristics such as age, gender, profession, medical history, sanitation, and hygiene information were recorded through a structured questionnaire. A stool sample was collected from each enrolled participant for testing with a rapid diagnostic test (RDT) for *V cholerae*. The RDT was carried out for immediate detection of cholera cases and reports were shared with the Early Warning and Alert Response System (EWARS) of WHO, a web-based system designed to enhance disease surveillance and outbreak detection in emergency settings for rapid response to control the disease. Samples were stored in Cary-Blair transport media in the field office at Cox’s Bazar at ambient temperature. Stored samples (in the Cary-Blair transport media at ambient temperature) can be preserved for more than 20 days [10]. Every week, the stored samples were transported in Cary-Blair transport media to the Mucosal Immunology and Vaccinology Laboratory at the icddr,b Dhaka. Upon receiving the specimens at the icddr,b laboratory, the samples were immediately processed for detection of *V cholerae* O1/O139, ETEC, and *Shigella* spp.

### Laboratory Procedures

For identification of *V cholerae*, specimens were streaked onto taurocholate-tellurite gelatin agar (TTGA) and incubated overnight at 37°C. Specimens were also incubated in alkaline peptone water for enrichment and incubated for an additional 18–24 hours and plated on TTGA [11]. Suspected colonies were serotyped with monoclonal antibody specific to *V cholerae O1* (Ogawa and Inaba) and O139 serogroups [12, 13]. For detection of ETEC, stool specimens were spread on MacConkey agar and incubated overnight at 37°C. Enterotoxigenic *E coli* was confirmed by multiplex polymerase chain reaction targeting the gene targets for ETEC heat-labile toxin and heat-stable toxin in lactose-fermenting colonies [14]. For detection of *Shigella* spp, specimens were streaked onto Salmonella Shigella agar, and then incubated overnight at 37°C, followed by systematic biochemical and serological testing methods (Denka Seiken Co., Ltd.).

### Statistical Analysis

We analyzed the relationship between enteric pathogens (*V cholerae*, ETEC, *Shigella* spp) detected in diarrheal patients among the FDMN and the host community populations and host sociodemographic factors. The demographic characteristics (age, gender, literacy status, household numbers, and water and sanitation practices) and clinical history of patients were analyzed through descriptive statistics. We performed Pearson χ ^2^ test to assess the statistical significance between categorical variables. To determine the independent factors associated with different enteric pathogens, we estimated crude odds ratios (ORs) using logistic regression. The factors crudely associated (*P* ≤.10) with enteric pathogens were fitted as independent variables in multiple regression models to adjusted ORs. All tests were performed as 2-tailed, and *P* < .05 was considered as the margin of statistical significance. The analyses were carried out by the statistical software R (version 3.6.1).

## RESULTS

Between 2017 and 2019, a total of 8134 participants were recruited into this study. Of these, 4881 were FDMNs and 3253 were from the Bangladeshi host community. Gender distribution was similar in FDMN and host community. Among the study population, 2752 (56.4%) were under 5 in the FDMN children and 1988 (61.1%) were Bangladeshi host population children who visited health facilities during the study period. Approximately 60% participants among FDMNs and 56% among Bangladeshi host population visited health facilities for diarrhea in the month of April–June/September–November in each year between 2017 and 2019.

Most of the diarrheal patients (FDMNs 94.4% and Bangladeshi 95.5%) suffered from diarrhea with prepresentation histories of 3 days or less. The FDMN participants were mostly illiterate (n = 3171 [78.5%]); the rate was lower (n = 1333 [48.3%]) among the host population participants. Among FDMNs participants, 87.9% used tube well, 94.6% used latrine, and 90.1% used soap after defecation; whereas in the participants from host community, 98.4% patients used tube well and latrine, and 79.2% used soap after defecation. Approximately 5.3% FDMN participants and 4.4% host population participants presented with severe dehydration. Approximately 58% FDMNs participants (n = 2827) and 66.5% participants from host population (n = 2164) visited health facilities with fever during diarrheal episodes. Among the tested samples, 0.7% *V cholerae*, 1.5% *Shigella* spp, and 4.9% ETEC were isolated from the FDMN participants, whereas 1.2%, 1.1%, and 1.8% were from the host community participants, respectively (Table 1). There were no differences in the rates of isolation of *Shigella* between the participants of FDMNs and host population (*P* > .05), but significant differences were seen between the 2 communities in case of isolation rates of *V cholerae* (OR = 0.50; 95% confidence interval [CI], 0.31–0.80; *P* = .004) and ETEC (OR = 2.85; 95% CI, 2.12–3.83; *P* < .001).

**Table 1.** Baseline Characteristics of Study Participants

Risk Factors	Labels	FDMN (%)	Host Population	*P* Value
Time (days)	0–180	1387 (28.4)	1004 (30.9)	
	181–365	924 (18.9)	601 (18.5)	
	366–545	774 (15.9)	600 (18.4)	
	546–730	1111 (22.8)	542 (16.7)	
	731–812	685 (14)	506 (15.6)	<.001
Duration of diarrhea (days)	0–3	4610 (94.4)	3106 (95.5)	
	4+	271 (5.6)	146 (4.5)	.038
Number of purging (times)	0–10	2344 (48)	1456 (44.8)	
	11–20	2355 (48.2)	1583 (48.7)	
	21+	182 (3.7)	214 (6.6)	<.001
Nature of stool	Watery	4823 (98.8)	3205 (98.5)	
	Bloody/semisolid/solid	58 (1.2)	48 (1.5)	NS
Sex	Female	2556 (52.4)	1745 (53.6)	
	Male	2325 (47.6)	1508 (46.4)	NS
Age (years)	0–4,	2752 (56.4)	1988 (61.1)	
	5+	2129 (43.6)	1265 (38.9)	<.001
Literate	Yes	870 (21.5)	1429 (51.7)	<.001
Family member	1–4	1665 (36.7)	1163 (38.5)	
	5+	2875 (63.3)	1855 (61.5)	NS
Tube well use	Yes	3554 (87.9)	2719 (98.4)	<.001
Latrine use	Yes	3824 (94.6)	2718 (98.4)	<.001
Soap use	Yes	3639 (90.1)	2188 (79.2)	<.001
Severe dehydration	Yes	259 (5.3)	144 (4.4)	NS
Vomiting	Yes	2385 (48.9)	1633 (50.2)	NS
Fever	Yes	2827 (57.9)	2164 (66.5)	<.001
*Vibrio cholerae* ^+^	Yes	33 (0.7)	40 (1.2)	.013
*Shigella* ^+^	Yes	72 (1.5)	37 (1.1)	NS
ETEC^+^	Yes	237 (4.9)	58 (1.8)	<.001

Abbreviations: ETEC, enterotoxigenic *Escherichia coli*; FDMN, Forcibly Displaced Myanmar Nationals; NS, not significant at 5% level of size.

### Cholera

Among the collected samples, 8077 were tested with RDT. The RDT test revealed 6.7% positivity (n = 328) from FDMNs participants and 5.6 % (n = 183) from the host community participants. Microbiological culture was done for 8134 samples and a total of 73 (0.9%) *V cholerae* were isolated by culture. Approximately 93% cholera cases were identified in the FDMN population during the months of April–June/September–November. Presentation with fever was similar in both of the communities (Supplementary Table 1). Among the FDMN participants, cholera detection was approximately 9 times higher in the period April–June/September–November in comparison to noncholera cases (OR = 8.77; 95% CI, 2.63–54.42; *P* = .003). In case of the participants among host community, cholera detection rate was approximately 5 times higher during the period of April–June/September–November between 2017 and 2019 (OR = 4.78; 95% CI, 2–14.14; *P* = .001). Among the FDMN participants, confirmed cholera cases had odds of severe dehydration, which was 2 times higher (OR = 2.10; 95% CI, 0.61–5.49; *P* = .171), and chance of vomiting was 2 times higher in comparison to noncases (OR = 2.28; 95% CI, 1.07–5.26; *P* = .04). On the other hand, among the host community participants, the chance of vomiting was 3 times higher (OR = 2.82; 95% CI, 1.32–6.72; *P* = .011) in cholera cases. Age >5 years was a significant (OR = 3.53; 95% CI, 1.72–7.78; *P* = .001) risk factor for cholera in cases among host community participants. Literacy was a protective factor for cholera in cases among FDMN participants (Table 2).

**Table 2.** Risk Factors Associated With Cholera in the Entire Study Population

Risk Factors	COR	*P* Value	AOR	*P* Value
FDMN				
Seasonal pattern (April–June/September–November)	9.33 (2.22–39.21)	.002	8.77 (2.63–54.42)	.003
Duration of diarrhea: 4+ days	0.58 (0.08–4.31)	.598	NA	NA
Bloody/semisolid/solid	0 (0, Inf)	.987	NA	NA
Age: 5+ years	1.29 (0.63–2.65)	.481	NA	NA
Male	0.96 (0.47–1.97)	.915	NA	NA
Literate	0.36 (0.08–1.55)	.172	NA	NA
Family member 5+	1.1 (0.49–2.46)	.825	NA	NA
Tube well use	0.87 (0.26–2.94)	.82	NA	NA
Latrine use	1 820 492.860, Inf)	.984	NA	NA
Soap use	2.32 (0.31–17.31)	.411	NA	NA
Severe dehydration	2.77 (0.96–8)	.06	2.1 (0.61–5.49)	.171
Vomiting	2.45 (1.12–5.37)	.025	2.28 (1.07–5.26)	.04
Fever	0.83 (0.4–1.7)	.611	NA	NA
Host Community				
Seasonal pattern (April–June/September–November)	5.52 (2.16–14.12)	0	4.78 (2–14.14)	.001
Duration of diarrhea: 4+ days	1.74 (0.53–5.71)	.361	NA	NA
Bloody/semisolid/solid	0 (0, Inf)	.982	NA	NA
Age: 5+ years	2.96 (1.54–5.69)	.001	3.53 (1.72–7.78)	.001
Male	0.95 (0.51–1.77)	.863	NA	NA
Literate	1.34 (0.67–2.66)	.407	NA	NA
Family member 5+	1.15 (0.57–2.34)	.696	NA	NA
Tube well use	538 742.5 (0, Inf)	.983	NA	NA
Latrine use	0.53 (0.07–3.95)	.534	NA	NA
Soap use	2.74 (0.83–8.98)	.097	2.18 (0.77–9.15)	.202
Severe dehydration	1.77 (0.54–5.8)	.348	NA	NA
Vomiting	3.01 (1.47–6.18)	.003	2.82 (1.32–6.72)	.011
Fever	0.61 (0.33–1.14)	.124	NA	NA

Abbreviations: AOR, adjusted odds ratio; COR, crude odds ratio; FDMN, Forcibly Displaced Myanmar Nationals; Inf, infinite; NA, not applicable.

NOTES: CORs, AORs, and *P* values are generated using logistic regression. For the adjusted model, we selected variables only for those which had an association with cholera with *P* ≤.10 in the crude analysis.

### Shigellosis

Among all stool samples, 109 (1.3%) were found to be positive for *Shigella* by culture. Among the detected cases, 84% were *Shigella flexneri*; and 7%, 5%, and 2% were *Shigella sonnei, Shigella boydii*, and *Shigella dysentery*, respectively. Among the *Shigella*-positive diarrheal patients, approximately 82% cases in FDMN participants and 63% cases in host community participants occurred during the month of April–June/September–November. Among the *Shigella* in FDMN participants, approximately 67% were female and 33% were male. The age distribution of the *Shigella* cases in FDMN participants was 35.8%, 14.9%, and 49.3% for the under 5 children, adolescents (5–14 years), and other patients (15 and above), respectively. On other hand, use of soap after defecation in the host community participants was 64.7%, whereas the proportion was 95.2% among FDMN participants. The majority of the shigellosis patient complained about fever in both the communities (Supplementary Table 2). The odds of occurrence of diarrhea due to *Shigella* among FDMN participants during April–June/September–November was 2 times higher (OR = 2.48; 95% CI, 1.35–4.94; *P* = .005). The odds of isolation of *Shigella* cases were approximately 11 times (OR = 10.87; 95% CI, 4.88–22.99; *P* = 0) higher when bloody stools were tested in FDMN populations. On the other hand, *Shigella* detection rate was approximately 26 times higher in case of bloody stool among the participant of host population (OR = 25.71, 95% CI, 5.27–97.73, *P* = 0). The FDMN participants in the >5-year age range were more prone to *Shigella* infection and the rate was approximately 3 times (OR = 2.54; 95% CI, 1.41–4.61; *P* = .002) higher (Table 3).

**Table 3.** Risk Factors Associated With Shigellosis in the Entire Study Population

Risk Factors	COR	*P* Value	AOR	*P* Value
FDMN				
Season pattern (April–June/September–November)	3.07 (1.64–5.74)	0	2.48 (1.35–4.94)	.005
Duration of diarrhea: 4+ days	1.69 (0.72–3.94)	.227	-	-
Bloody/semisolid/solid	22.59 (11.35–44.98)	0	10.87 (4.88–22.99)	0
Age: 5+ years	2.34 (1.42–3.87)	.001	2.54 (1.41–4.61)	.002
Male	0.53 (0.32–0.89)	.016	0.5 (0.28–0.85)	.012
Literacy	0.61 (0.18–2.06)	.422	NA	NA
Family members 5+	1 (0.53–1.9)	.989	NA	NA
Tube well use	1.3 (0.3–5.62)	.721	NA	NA
Latrine use	1.14 (0.15–8.51)	.901	NA	NA
Soap use	2.21 (0.3–16.52)	.439	NA	NA
Severe dehydration	1.13 (0.41–3.14)	.809	NA	NA
Vomiting	0.62 (0.38–1.02)	.059	0.67 (0.39–1.11)	.125
Fever	1.15 (0.7–1.88)	.585	NA	NA
OCV received	0.41 (0.24–0.69)	.001	0.29 (0.16–0.52)	0
Host Community				
Seasonal pattern (April–June/September–November)	1.32 (0.66–2.63)	.432	NA	NA
Duration of diarrhea: 4+ days	2.01 (0.61–6.66)	.251	NA	NA
Bloody/semisolid/solid	24.14 (10.32–56.46)	0	25.71 (5.27–97.73)	0
Age: 5+ years	2.38 (1.21–4.71)	.012	2.11 (0.74–6.38)	.164
Male	0.68 (0.34–1.36)	.275	NA	NA
Literacy	0.39 (0.14–1.1)	.075	0.4 (0.13–1.11)	.094
Family members 5+	0.58 (0.27–1.24)	.159	NA	NA
Tube well use	727 889.49 (0, Inf)	.989	NA	NA
Latrine use	728 159.07 (0, Inf)	.989	NA	NA
Soap use	0.48 (0.18–1.3)	.148	NA	NA
Severe dehydration	2.04 (0.62–6.76)	.241	NA	NA
Vomiting	0.66 (0.33–1.3)	.228	NA	NA
Fever	1.46 (0.68–3.13)	.329	NA	NA

Abbreviations: AOR, adjusted odds ratio; COR, crude odds ratio; FDMN, Forcibly Displaced Myanmar Nationals; Inf, infinite; NA, not applicable; OCV, oral cholera vaccines.

NOTES: CORs, AORs, and *P* values are generated using logistic regression. For the adjusted model, we selected variables only for those which had an association with shigellosis with *P* ≤.10 in the crude analysis.

### Enterotoxigenic *Escherichia coli* Infection

A total of 295 (3.6%) ETEC infections were detected from the 8134 enrolled patients. Approximately 75% of ETEC cases of FDMN participants occurred during the month of April–June/September–November. Among all ETEC-positive FDMN participants, 53.4% were children <5 years, 6.9% were children and adolescents aged 5–14 years, and 39.7% were aged 15 and above. On the other hand, from the host community participants, 44.6% were children <5 years and 55.4% were aged 15 years and above, but there was no ETEC diarrhea in the 5–14 age group (Supplementary Table 3). The surveillance shows that chances of ETEC-confirmed cases was approximately 2 times higher (OR = 1.58; 95% CI, 1.16–2.18; *P* = .004) during the months of April–June and September–November among FDMN participants. Among the host population, those in the >5-year age group were at higher risk for ETEC diarrhea (OR = 2.39; 95% CI, 1.20–4.89; *P* = .013) (Table 4).

**Table 4.** Risk Factors Associated With ETEC Infection in the Entire Study Population

Risk Factors	COR	*P* Value	AOR	*P* Value
FDMN				
Seasonal pattern (April–June/September–November)	2 (1.48–2.7)	0	1.58 (1.16–2.18)	.004
Duration of diarrhea: 4+ days	1.01 (0.57–1.79)	.975	NA	NA
Bloody/semisolid/solid	0.35 (0.05–2.53)	.297	NA	NA
Age: 5+ years	1.13 (0.87–1.48)	.357	NA	NA
Male	0.81 (0.62–1.06)	.124	NA	NA
Literacy	0.88 (0.6–1.3)	.514	NA	NA
Family member 5+	1.07 (0.8–1.43)	.663	NA	NA
Tube well use	1.54 (0.89–2.69)	.126	NA	NA
Latrine use	0.81 (0.43–1.51)	.505	NA	NA
Soap use	1.79 (0.94–3.42)	.079	1.63 (0.92–3.28)	.129
Severe dehydration	1.64 (1.01–2.66)	.047	1.52 (0.77–2.67)	.181
Vomiting	0.99 (0.76–1.29)	.959	NA	NA
Fever	0.91 (0.7–1.18)	.469	NA	NA
Host Community				
Seasonal pattern (April–June/September–November)	1.96 (1.09–3.52)	.024	1.24 (0.632–2.488)	.534
Duration of diarrhea: 4+ days	0.38 (0.05–2.78)	.342	NA	NA
Bloody/semisolid/solid	0 (0, Inf)	.981	NA	NA
Age: 5+ years	1.98 (1.16–3.36)	.012	2.389 (1.208–4.869)	.013
Male	0.8 (0.47–1.37)	.425	NA	NA
Literacy	1.8 (0.89–3.64)	.1	NA	NA
Family members 5+	1.96 (0.99–3.88)	.054	1.406 (0.687–3.103)	.37
Tube well use	Inf^a^	NA	NA	NA
Latrine use	0.26 (0.06–1.11)	.069	0.214 (0.06–1.363)	.041
Soap use	1.27 (0.53–3.08)	.593	NA	NA
Severe dehydration	1.23 (0.38–3.97)	.734	NA	NA
Vomiting	1.33 (0.78–2.27)	.296	NA	NA
Fever	1.06 (0.6–1.87)	.829	NA	NA

Abbreviations: AOR, adjusted odds ratio; COR, crude odds ratio; ETEC, enterotoxigenic *Escherichia coli*; FDMN, Forcibly Displaced Myanmar Nationals; Inf, infinite; NA, not applicable.

NOTES: CORs, AORs, and *P* values are generated using logistic regression. For the adjusted model, we selected variables only for those which had an association with ETEC infection with *P* ≤.10 in the crude analysis.

^a^The estimate was not stable due low sample size.

## DISCUSSION

The study describes the enteric disease surveillance carried out among FDMN participants and host community participants in Cox’s Bazar, with an emphasis on cholera, ETEC and *Shigella* spp. More than 8000 samples were collected from patients, 60% of whom were from FDMN participants and 40% of whom were from the host community. Isolation of ETEC was highest in both communities, which includes FDMN participants (~70%) and host communities’ participants (~44%). The increased number of care seeking for diarrheal diseases was significantly associated with seasonality, and more than half of all visits occurred in the month of April–June/September–November between 2017 and 2019 for both communities. This seasonal variability demonstrates similar findings from a nationwide surveillance in Bangladesh carried out between 2014 and 2018 [9]. Moreover, the system can pick up these changes and can be notified for early decision making. Participants with children <5 years of age for both communities were highest (FDMN participants, 56.4%; host community participants, 61.1%). Participants from host community were more used to using tube well and latrine than the FDMN participants, but they lagged behind in soap use after defecation. This may be the outcome of robust promotion of WaSH materials by different organizations after the influx FDMNs in the makeshift camps [15]. Stool culture reports showed that cholera was relatively higher among the host community patients, but, comparatively, *Shigella* diarrhea and ETEC diarrhea occurred more often among FDMN patients. More than two thirds of the *Shigella* cases were female, and approximately half of the population were aged 15 years and above. A similar pattern was observed in a study on shigellosis conducted in Taiwan [16]. However, another study conducted in 6 Asian countries revealed that children <5 years old were mostly affected by *Shigella* [17].

The major strength of the surveillance team was its expertise and extensive experience with nationwide enteric diseases surveillance including Cox’s Bazar from 2014 onwards. This surveillance has been ongoing in 10 district hospitals since 2014 and was extended to 12 more district and subdistrict hospitals. All of the surveillance sites were established within a 1-hour distance from the housing of the FDMNs and host community living in the camp areas. The surveillance was carried out in collaboration with the government of Bangladesh and national and international NGO hospitals. Moreover, the surveillance was continuously monitored by icddr,b and healthcare facility supervisors, and based on the findings of the surveillance report, the policymakers from government and nongovernment organizations accepted assessments about cholera vaccination among FDMN participants and, subsequently, the host community to prevent a sudden upsurge of cholera or disease outbreak. The surveillance results were also used for EWARS of WHO to contain cholera in the densely populated areas that lacked proper water, sanitation, and hygiene facilities.

The major limitation of the study was a language barrier for communicating with the FDMNs. The hurdle was overcome by recruiting surveillance staff, such as medical technologist supervisors and medical officers, from the local community who were familiar with FDMNs’ mother tongue and local language. However, a large number of OCVs were delivered to FDMNs and the host community, but vaccination information could not be used in this analysis because the source of information (vaccination card) was not available during data collection.

## CONCLUSIONS

In conclusion, this study provides critical insights for the control of diarrheal diseases due to specific enteric pathogens among FDMN as well as the host community. Continuation and expansion of the surveillance will play a crucial role for early detection of the cases to identify any cholera epidemics or outbreaks among the host community as well as FDMNs. In addition, alignment of the surveillance data with the EWARS will be a comprehensive strategy for diarrheal disease control in Bangladesh, which can be replicated for similar settings around the globe.

## Supplementary Data

Supplementary materials are available at *The Journal of Infectious Diseases* online. Consisting of data provided by the authors to benefit the reader, the posted materials are not copyedited and are the sole responsibility of the authors, so questions or comments should be addressed to the corresponding author.

jiab453_suppl_Supplementary_Table_S1

jiab453_suppl_Supplementary_Table_S2

jiab453_suppl_Supplementary_Table_S3

## Notes

***Acknowledgments.*** International Centre for Diarrhoeal Disease Research Bangladesh (icddr,b) is grateful to the Government of Bangladesh, Canada, Sweden, and the United Kingdom for providing core/unrestricted support for this publication. In addition, we thank the Bill & Melinda Gates Foundation (INV-033599) for support to publish this article.

***Financial support.*** This research was funded by the United Nations Children’s Fund (UNICEF) (BGD/PCA201840/PD2019319).

***Potential conflicts of interest.*** All authors: No reported conflicts. All authors have submitted the ICMJE Form for Disclosure of Potential Conflicts of Interest.
