# Supplementary material for: Acute Watery Diarrhea Surveillance During the Rohingya Crisis 2017–2019 in Cox’s Bazar, Bangladesh
Source: J Infect Dis. 2021 Sep 16;224(Suppl 7):S717–24. doi: 10.1093/infdis/jiab453 (PMC8687071; doi:10.1093/infdis/jiab453)
Supplement: jiab453_suppl_Supplementary_Table_S1 [file jiab453_suppl_supplementary_table_s1.docx]

|  |  |  | **Country** | |  | |
| --- | --- | --- | --- | --- | --- | --- |
| **Factors** | **Labels** |  | **FDMN: n (%)** | **Host community: n (%)** | **P value** |  |
| Time | 0-180 Days |  | 8 (26.7) | 8 (20) |  |  |
|  | 181-365 Days |  | 3 (10) | 7 (17.5) |  |  |
|  | 366-545 Days |  | 5 (16.7) | 3 (7.5) |  |  |
|  | 546-730 Days |  | 7 (23.3) | 15 (37.5) |  |  |
|  | 731-812 Days |  | 7 (23.3) | 7 (17.5) | 0.455 |  |
| Season (April-June/Sept-Nov) | No |  | 2 (6.7) | 5 (12.5) |  |  |
|  | Yes |  | 28 (93.3) | 35 (87.5) | 0.687 |  |
| Duration of Diarrhoea | 0-3 days |  | 29 (96.7) | 37 (92.5) |  |  |
|  | 4+ days |  | 1 (3.3) | 3 (7.5) | 0.824 |  |
| Number of purging | 0-10 times |  | 12 (40) | 11 (27.5) |  |  |
|  | 11-20 times |  | 16 (53.3) | 25 (62.5) |  |  |
|  | 21+ times |  | 2 (6.7) | 4 (10) | 0.526 |  |
| Sex | Female |  | 16 (53.3) | 22 (55) |  |  |
|  | Male |  | 14 (46.7) | 18 (45) | 1.000 |  |
| Age | 0-4, years |  | 15 (50) | 14 (35) |  |  |
|  | 5-14, years |  | 5 (16.7) | 5 (12.5) |  |  |
|  | 15+, years |  | 10 (33.3) | 21 (52.5) | 0.278 |  |
| Literate | No |  | 20 (90.9) | 14 (41.2) |  |  |
|  | Yes |  | 2 (9.1) | 20 (58.8) | 0.001 |  |
| Family Member | 1-4, Members |  | 9 (34.6) | 12 (35.3) |  |  |
|  | 5+, Members |  | 17 (65.4) | 22 (64.7) | 1.000 |  |
| Tube-well Use | No |  | 3 (13.6) | 0 (0) |  |  |
|  | Yes |  | 19 (86.4) | 34 (100) | 0.108 |  |
| Latrine Use | No |  | 0 (0) | 1 (2.9) |  |  |
|  | Yes |  | 22 (100) | 33 (97.1) | 1.000 |  |
| Soap Use | No |  | 1 (4.5) | 3 (8.8) |  |  |
|  | Yes |  | 21 (95.5) | 31 (91.2) | 0.940 |  |
| Severe Dehydration | No |  | 26 (86.7) | 37 (92.5) |  |  |
|  | Yes |  | 4 (13.3) | 3 (7.5) | 0.687 |  |
| Vomiting | No |  | 9 (30) | 10 (25) |  |  |
|  | Yes |  | 21 (70) | 30 (75) | 0.846 |  |
| Fever | No |  | 14 (46.7) | 18 (45) |  |  |
|  | Yes |  | 16 (53.3) | 22 (55) | 1.000 |  |

## **Supplementary Table 1: Characteristics of cholera patients by population**

**Note:** *P values are generated using Chi-square test (P values may not accurate for cell frequency <5)*
