# Supplementary material for: Acute Watery Diarrhea Surveillance During the Rohingya Crisis 2017–2019 in Cox’s Bazar, Bangladesh
Source: J Infect Dis. 2021 Sep 16;224(Suppl 7):S717–24. doi: 10.1093/infdis/jiab453 (PMC8687071; doi:10.1093/infdis/jiab453)
Supplement: jiab453_suppl_Supplementary_Table_S2 [file jiab453_suppl_supplementary_table_s2.docx]

## **Supplementary Table 2: Characteristics of Shigellosis patients by population**

|  |  | **Country** | |  |
| --- | --- | --- | --- | --- |
| **Factors** | **Labels** | **FDMN: n (%)** | **Host community: n (%)** | **P value** |
| Time | 0-180 Days | 53 (79.1) | 21 (60) |  |
|  | 181-365 Days | 6 (9) | 5 (14.3) |  |
|  | 366-545 Days | 2 (3) | 2 (5.7) |  |
|  | 546-730 Days | 4 (6) | 7 (20) |  |
|  | 731-812 Days | 2 (3) | 0 (0) | 0.114 |
| Season (April-June/Sept-Nov) | No | 12 (17.9) | 13 (37.1) |  |
|  | Yes | 55 (82.1) | 22 (62.9) | 0.057 |
| Duration of Diarrhea | 0-3 days | 61 (91) | 32 (91.4) |  |
|  | 4+ days | 6 (9) | 3 (8.6) | 1.000 |
| Number of purging | 0-10 times | 41 (61.2) | 21 (60) |  |
|  | 11-20 times | 24 (35.8) | 13 (37.1) |  |
|  | 21+ times | 2 (3) | 1 (2.9) | 0.991 |
| Sex | Female | 45 (67.2) | 22 (62.9) |  |
|  | Male | 22 (32.8) | 13 (37.1) | 0.830 |
| Age | 0-4, years | 24 (35.8) | 14 (40) |  |
|  | 5-14, years | 10 (14.9) | 4 (11.4) |  |
|  | 15+, years | 33 (49.3) | 17 (48.6) | 0.854 |
| Literate | No | 18 (85.7) | 12 (70.6) |  |
|  | Yes | 3 (14.3) | 5 (29.4) | 0.461 |
| Family Member | 1-4, Members | 15 (36.6) | 14 (51.9) |  |
|  | 5+, Members | 26 (63.4) | 13 (48.1) | 0.320 |
| Tube-well Use | No | 2 (9.5) | 0 (0) |  |
|  | Yes | 19 (90.5) | 17 (100) | 0.564 |
| Latrine Use | No | 1 (4.8) | 0 (0) |  |
|  | Yes | 20 (95.2) | 17 (100) | 1.000 |
| Soap Use | No | 1 (4.8) | 6 (35.3) |  |
|  | Yes | 20 (95.2) | 11 (64.7) | 0.046 |
| Severe Dehydration | No | 63 (94) | 32 (91.4) |  |
|  | Yes | 4 (6) | 3 (8.6) | 0.936 |
| Vomiting | No | 42 (62.7) | 21 (60) |  |
|  | Yes | 25 (37.3) | 14 (40) | 0.960 |
| Fever | No | 26 (38.8) | 9 (25.7) |  |
|  | Yes | 41 (61.2) | 26 (74.3) | 0.270 |

**Note:** *P values are generated using Chi-square test (P values may not accurate for cell frequency <5)*
