# Supplementary material for: Acute Watery Diarrhea Surveillance During the Rohingya Crisis 2017–2019 in Cox’s Bazar, Bangladesh
Source: J Infect Dis. 2021 Sep 16;224(Suppl 7):S717–24. doi: 10.1093/infdis/jiab453 (PMC8687071; doi:10.1093/infdis/jiab453)
Supplement: jiab453_suppl_Supplementary_Table_S3 [file jiab453_suppl_supplementary_table_s3.docx]

|  | **Country** | |  |  |
| --- | --- | --- | --- | --- |
| **Factors** | **Labels** | **FDMN: n (%)** | **Host community: n (%)** | **P value** |
| Time | 0-180 Days | 76 (32.8) | 23 (41.1) |  |
|  | 181-365 Days | 41 (17.7) | 7 (12.5) |  |
|  | 366-545 Days | 31 (13.4) | 10 (17.9) |  |
|  | 546-730 Days | 58 (25) | 16 (28.6) |  |
|  | 731-812 Days | 26 (11.2) | 0 (0) | 0.067 |
| Season (April-June/Sept-Nov) | No | 59 (25.4) | 16 (28.6) |  |
|  | Yes | 173 (74.6) | 40 (71.4) | 0.756 |
| Duration of Diarrhea | 0-3 days | 219 (94.4) | 55 (98.2) |  |
|  | 4+ days | 13 (5.6) | 1 (1.8) | 0.397 |
| Number of purging | 0-10 times | 117 (50.4) | 32 (57.1) |  |
|  | 11-20 times | 103 (44.4) | 21 (37.5) |  |
|  | 21+ times | 12 (5.2) | 3 (5.4) | 0.640 |
| Sex | Female | 133 (57.3) | 33 (58.9) |  |
|  | Male | 99 (42.7) | 23 (41.1) | 0.947 |
| Age | 0-4, years | 124 (53.4) | 25 (44.6) |  |
|  | 5-14, years | 16 (6.9) | 0 (0) |  |
|  | 15+, years | 92 (39.7) | 31 (55.4) | 0.028 |
| Literate | No | 136 (80.5) | 12 (34.3) |  |
|  | Yes | 33 (19.5) | 23 (65.7) | 0 |
| Family Member | 1-4, Members | 73 (35.3) | 11 (24.4) |  |
|  | 5+, Members | 134 (64.7) | 34 (75.6) | 0.222 |
| Tube-well Use | No | 14 (8.3) | 0 (0) |  |
|  | Yes | 155 (91.7) | 35 (100) | 0.162 |
| Latrine Use | No | 11 (6.5) | 2 (5.7) |  |
|  | Yes | 158 (93.5) | 33 (94.3) | 1.000 |
| Soap Use | No | 10 (5.9) | 6 (17.1) |  |
|  | Yes | 159 (94.1) | 29 (82.9) | 0.057 |
| Severe Dehydration | No | 213 (91.8) | 53 (94.6) |  |
|  | Yes | 19 (8.2) | 3 (5.4) | 0.663 |
| Vomiting | No | 119 (51.3) | 24 (42.9) |  |
|  | Yes | 113 (48.7) | 32 (57.1) | 0.325 |
| Fever | No | 103 (44.4) | 18 (32.1) |  |
|  | Yes | 129 (55.6) | 38 (67.9) | 0.129 |

## **Supplementary Table 3: Characteristics of ETEC patients by population**

**Note:** *P values are generated using Chi-square test (P values may not accurate for cell frequency <5)*
